# Prediction of Degreening Velocity of Broccoli Buds Using Hyperspectral Camera Combined with Artificial Neural Networks

**DOI:** 10.3390/foods9050558

**Published:** 2020-05-02

**Authors:** Yoshio Makino, Yumi Kousaka

**Affiliations:** Graduate School of Agricultural and Life Sciences, The University of Tokyo, 1-1-1, Yayoi, Bunkyo-ku, Tokyo 113-8657, Japan; yumi.kousaka@gmail.com

**Keywords:** *Brassica oleracea* var. *italica*, chlorophyll, mathematical model, nondestructive analysis, shelf life, spectroscopy, statistical analysis, vegetable

## Abstract

Developing a noninvasive technique to estimate the degreening (loss of green color) velocity of harvested broccoli was attempted. Loss of green color on a harvested broccoli head occurs heterogeneously. Therefore, hyperspectral imaging technique that stores spectral reflectance with spatial information was used in the present research. Using artificial neural networks (ANNs), we demonstrated that the reduction velocity of chlorophyll at a site on a broccoli head was related to the second derivative of spectral reflectance data at 15 wavelengths from 405 to 960 nm. The reduction velocity was predicted using the ANNs model with a correlative coefficient of 0.995 and a standard error of prediction of 5.37 × 10^−5^ mg·g^−1^·d^−1^. The estimated reduction velocity was effective for predicting the chlorophyll concentration of broccoli buds until 7 d of storage, which was established as the maximum time for maintaining marketability. This technique may be useful for nondestructive prediction of the shelf life of broccoli heads.

## 1. Introduction

Broccoli (*Brassica oleracea* var. *italica*) is one of the main vegetables with 26.5 Mt of the commodity harvested in 2018 in the world (FAOSTAT, broccoli + cauliflower [*Brassica oleracea* var. *botrytis*]) [1]. Since the early 1980s, broccoli has been reported as a food to depress cancer [2] since it includes sulforaphane [3,4], which is useful for depressing the growth of *Helicobacter pylori*, the main reason for gastric cancer [5]. However, loss of green color (degreening) is the main phenomenon that decreases the shelf life of harvested broccoli [6] and is induced by the reduction of pigment (chlorophyll) [7]. Loss of green color may cause a reduction of effects suitable for health-promotion by broccoli since chlorophyll is useful for reducing serum cholesterol in mammals [8,9]. Lipton & Harris [10] subjectively evaluated the appearance of broccoli heads by scales (nine levels), while Jacobssonet al. [11] chose a visual technique to assess the appearance of broccoli and determined a threshold that 30% of the buds had turned yellow. Objective measurement of the appearance of broccoli heads has also been conducted. Shewfelt et al. [6] noninvasively evaluated the appearance of six cultivars of harvested broccoli by two types of colorimeters. Ren et al. [12] proposed models for evaluating changes in broccoli color on the basis of values measured using a colorimeter, though Kasim et al. [13] measured the hue angle using a colorimeter to assess the color of broccoli packaged in several kinds of film pouches after use of 1-methylcyclopropene (1-MCP). However, Lipton & Harris [10] published that localization of degreening (loss of green color) was observed. This report indicated that it is difficult to select a colorimeter (point-based method) to measure loss of green color at local sites. In contrast, hyperspectral camera has been utilized in studies to measure pigment concentrations in forests as a tool for remote sensing [14]. This method has been used as a technique for evaluating food safety and quality control [15]. Hyperspectral imaging (spatial spectral reflectance data) was utilized by Qin et al. [16] to find citrus cankers and Ariana & Lu [17,18] to find internal defects and the appearance of whole pickles and cucumbers (*Cucumis sativus* L.). Ahn et al. [19] measured nutrient contents in some kinds of food products using hyperspectral signals based on deep neural networks. Feng et al. [20] evaluated the hygiene of sausage by measuring the spatial distribution of adenosine 5′-triphosphate using hyperspectral camera. Applications of a hyperspectral imaging camera for inspecting food quality and safety have been reported day by day. These studies indicated that hyperspectral imaging may be useful for finding loss of green color on broccoli heads because it provides both spectral and spatial data. For example, the chlorophyll content at leaf and canopy levels in forests was assumed by hyperspectral imaging as a remote sensing technique [21,22]. Xue and Yang [23] measured chlorophyll content in some kinds of leafy vegetables using hyperspectral camera. Kabakeriset al. [24] evaluated quality loss of broccoli by hyperspectral imaging.

In the current study, we tried to estimate the velocity of loss of green color of harvested broccoli by a more advanced method than that used in previous researches. Estimation of the velocity of loss of green color at random locations at the initiation of storage or transportation permits the grading of products according to their shelf lives. This grading is effective for determining the order of shipment and distribution as raw or frozen products. This method is effective for reduction of waste as well. According to the report [25], 34% of fruits and vegetables produced were wasted without consumption in the world. Reduction of the waste using the proposed grading method is equivalent to the increase of production. Near-infrared spectroscopy combined with artificial neural networks (ANNs) is useful for estimating the content of components in horticultural commodities [26]. The effectiveness of ANNs in food analysis has also been reported [27].

The objective of this research was to propose a technique for estimating the velocity of loss of green the color of harvested broccoli by ANNs and spectral reflectance on the basis of hyperspectral images.

## 2. Materials and Methods

### 2.1. Samples and Preparation

Heads of broccoli (Sakata Seed Corporation, cv. SK048) were harvested 1 day before experiments at a farmland in Fukaya (N: 36.209749, E: 139.215149, Saitama Pref., Japan). After harvest, the samples in cardboard boxes were carried to the laboratory at 5 °C within 24 h. Seventeen broccoli heads were selected as samples and the size of samples (height) were adjusted to 130 mm by trimming the main stems of the heads so that the aperture between the heads and the illumination rod remained constant. The samples were wrapped in macro-perforated low-density polyethylene pouches (eight perforations per pouch; diameter, 6 mm; thickness, 25 μm) and stored in a chamber at 5 °C and 70% relative humidity for the following experiments.

### 2.2. Determination of Spectral Data by the Hyperspectral Camera System

**Figure 1:**
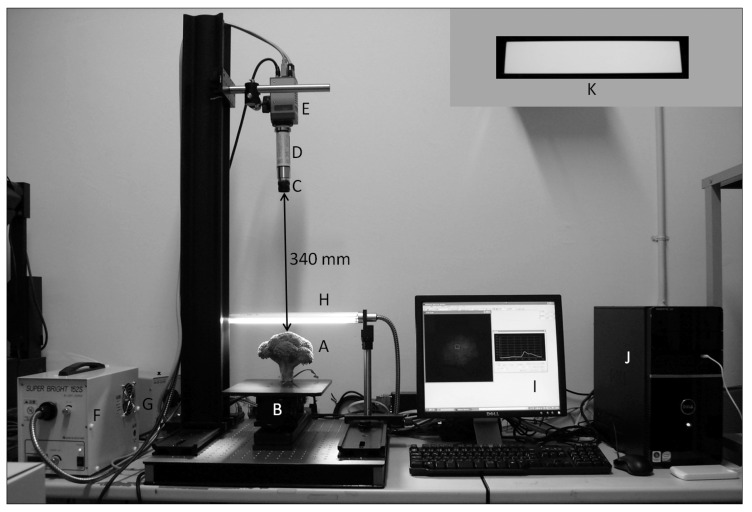
Overall view and description of the components in the hyperspectral camera system (JFE Techno-Research Corporation, Tokyo, Japan) [28]. A, Sample; B, Sample stage; C, Lens; D, Spectrograph; E, 12 bit CCD camera; F, 150 W Xe lamp; G, 150 W tungsten halogen lamp; H, 250 mm illumination rod; I, 17 inch monitor; J, desktop computer; K, 40 mm × 220 mm white reference.

The overall view and description of the parts of the hyperspectral camera system (JFE Techno-Research Corporation, Tokyo, Japan) are presented in Figure 1, the same as the previous report [28].

The prepared broccoli samples were stored at 5 °C in a chamber for 48 d. The spectral reflectance of the samples was determined by the system over the wavelength in the range of 380–1000 nm, wavelength resolution of 5 nm, and driving velocity of the stage of 0.41 mm·s^−1^. Intensity of light reflectance from a sample was transformed by recording the dark current image to 0 and from white standard image to 1. A SpectrumAnalyzer^®^ ver. 1.8.6 (JFE Techno-Research Corporation, Tokyo, Japan) was utilized for getting and analyzing the “hyper-cube data” [15] acquired using the system. Then it was assumed that the curvature effect on the broccoli head was too small to be ignored in the present study. The mass of each sample was determined during storage as well as the spectral reflectance.

### 2.3. Destructive Measurement of Chlorophyll Content in Broccoli

Pigment contents were related to reflectance values referred to the publication by Nicotra et al. [22]. The spectral reflectance of a broccoli head was measured before and after the following sampling treatments. Broccoli buds were sampled from around the center of the head and stored at −80 °C until use. The spectral reflectance at the sampling sites on the buds was determined by comparing the reflectance before and after the sampling. The hue angle (tan^−1^ (b*/a*)) was determined from the spectral reflectance that ranged from 380 to 780 nm by the method of the Commission International de l’Eclairage (CIE). The chlorophyll content (mg·g^−1^) was determined by measuring 80% acetone solution including chlorophyll prepared from bud samples using a UV-3600 spectrophotometer (Shimadzu Corporation, Kyoto, Japan) [29]. An equation to relate *C* to *H*° was proposed by simple regression analysis reported by Ren et al. [12].

### 2.4. Constraction of a Model to Predict the Velocity of Chlorophyll Reduction

Ten arbitrary 6 mm × 6 mm regions of interest (ROIs) were chosen from a broccoli head and the mean values of spectral reflectance of these ROIs was determined (spatial resolution: 0.00281 mm^2^ per pixel). The mean value at the same ROIs chosen above were collected from the start (0 d) to the end of storage (48 d). Eleven heads (110 ROIs) and six heads (60 ROIs) were used for the calibration and validation sets.

The chlorophyll contents were drawn in a scatter graph, and the changes over time were fitted using straight lines. According to the experimental data, the degradation velocity of chlorophyll during storage was presumed to be constant in the study. The degradation velocity can be expressed as follows:

*C_t_* = *C*_0_ + *kt*, (1)

The value of parameter *k* should be predicted from spectral reflectance at the start of storage. Then, the degreening velocity of broccoli buds during storage can be predicted using nondestructive analysis.

Calculations in this research were conducted using JMP^®^ 8.0.2 (SAS Institute Inc., Cary, North Carolina, USA), with the exception of the second derivative of spectral reflectance (δ^2^ *R*), which was calculated using Origin^®^ 7.5J (Lightstone Co., Tokyo, Japan). The wavelengths needed for construction of the equations were chosen by linear correlative coefficients between the second derivative of spectral reflectance (δ^2^ *R*) as the independent variables and *k* as an exemplar at each wavelength, using the 110 samples chosen for calibration. The linear correlative coefficient was determined as follows:

Equations were made using ANNs and the calibration values. The δ^2^ *R* values at some wavelengths were chosen as independent variables for ANN modeling.

ANNs use a non-linear fitting way and are considered to be useful for complicated objects such as biological organizations. JMP software applies plural approximation methods, such as the Gauss-Newton way, a type of logistic curve, as the activating function for ANNs. The ANNs contained an input layer, a hidden layer of neurons, and an output layer.

*X_ih_* = *A_h_* + ∑ [*j* = 1, *m*] *B_jh_*·*d_ij_*, (2)

*H_ih_* = 1/[1 + exp(−*X_ih_*)], (3)

(4) $${\hat{k}}_{i}=C+\sum \;[h=1,\;l]\;D_{h}\cdot H_{ih},$$

ANNs were conducted under the conditions as follows: the number of layers, 3; number of hidden nodes, 3; number of repeated calculations, 75; over fit penalty, 0.01; number of calculations, 1; standard of convergence, 0.5; and random number seed 274510453. ANN calculation can be reproduced under the conditions mentioned above.

### 2.5. Precision of a Proposed Equation

To calibrate the method, the standard error of calibration (SEC) and correlative coefficient of calibration were calculated using Equation (5), and used as indices to evaluate the fit of the proposed equation to the actual values.

(5) $$\mathrm{SEC}={\left[\{\sum \;[i=1,\;n]{\left(k_{i}-{\hat{k}}_{i}\right)}^{2}\}/(n-j-1)\right]}^{0.5},$$

Data of δ^2^ *R* for 60 samples at the 15 chosen wavelengths were utilized as the input data for validating the calibrated ANNs. Standard error of prediction (SEP) and bias, used as indicators for testing the approximation of a proposed equation to the actual values for validation, were checked using the predicted $\hat{k}$ values as output data:

(6) $$\bar{b}=(1/n)\sum \;[i=1,\;n]b_{i},$$

(7) $$(b_{i}=k_{i}-{\hat{k}}_{i}),$$

(8) $$\mathrm{SEP}={\left[\{\sum \;[i=1,\;n]{\left(b_{i}-{\bar{b}}_{i}\right)}^{2}\}/(n-1)\right]}^{0.5},$$

Bias is the mean difference between the predicted and actual values. The correlative coefficient of validation was determined by conventional methods from the validation data. SEP, the correlative coefficient of validation, was the index used for checking the approximation of a proposed equation to the measured values for validation.

## 3. Results and Discussion

**Figure 2:**
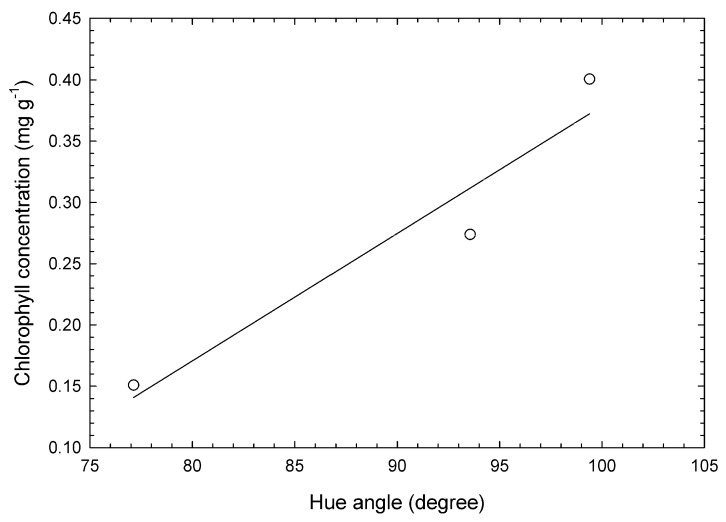
Relationship between hue angle and chlorophyll concentration in broccoli buds. The open circles and full line are the actual values and single regression line (*C* = 0.0104*H*° − 0.6613). Bias was 2.12 × 10^−4^ mg·g^−1^, standard error of calibration 2.79 × 10^−2^ mg·g^−1^, relative percent difference 4.48, and correlative coefficient of validation 0.962.

The relationship between *H*° and chlorophyll concentration was approximated by the calibration line as shown in Figure 2. The observed minimum *H*° in the current study was 80.1. Therefore, the *C* value was never a negative value.

The chlorophyll concentration of the broccoli buds decreased linearly over time in the current study. Van Boekel [30] and Ren et al. [12] approximated the changes in chlorophyll concentrations using logarithmic functions. However, the degreening velocity was determined as the slope of the experimental data calculated by linear regression analysis using Equation (1). The velocity during storage was attempted to predict using hyperspectral images on the initial day of storage in the present study.

**Figure 3:**
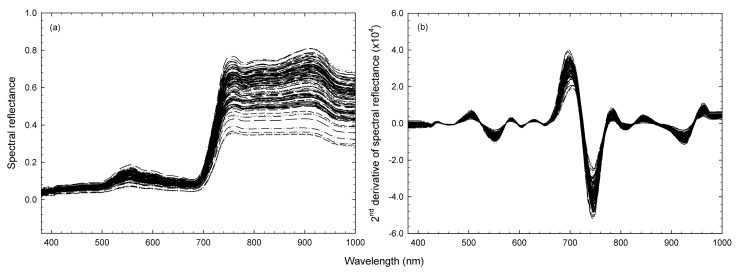
(**a**) Raw and (**b**) second derivatives of spectral reflectance in the range of 380 and 1000 nm from 11 broccoli heads (110 regions of interest).

The raw and second derivatives of spectral reflectance in the range of 380 and 1000 nm from 11 broccoli heads (110 ROIs) are shown in Figure 3. This pretreatment was conducted to eliminate the influence of baseline on spectral reflectance [31]. The pretreated data was used for constructing an ANN calibration model.

**Figure 4:**
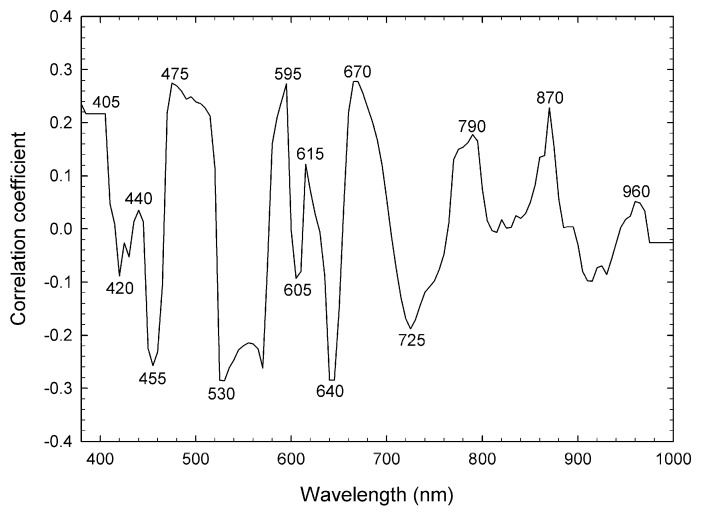
Correlative coefficients between the chlorophyll degradation velocity and reflectance at 5 nm wavelength bands between 380 and 1000 nm.

The correlative coefficients at every wavelength between *k* and δ^2^ *R* are shown in Figure 4. Fifteen of the wavelengths with a high correlative coefficient (absolute value) and without multicollinearity were chosen (Figure 4). The δ^2^ *R* values at six wavelengths correlated negatively with the *k* value, while the δ^2^ *R* values at nine wavelengths correlated positively with the *k* value, and thus, were chosen as independent variables (Figure 4). Peiris et al. [32] predicted the total soluble solid (TSS) of tomatoes using the second derivative of spectral absorbance in the range of 780–980 nm. Therefore, the range of absorption spectra in this research may be related to TSS, which is composed mainly of sucrose, fructose, and glucose. These sugars stimulate metabolic activity because they are associated with glycolysis, which contributes to the generation of adenosine 5′-triphosphate needed for many biological reactions. Therefore, reflectance may be associated with the *k* value that represents the degradation velocity of chlorophyll. According to the Merck Index [33], reflectance between 405 and 670 nm may be associated with chlorophyll *a* and *b*. However, the correlative coefficients between reflectance values at selected wavelengths and *k* values were not as high. This suggests that chlorophyll concentration at the start of storage did not affect the deterioration velocity of chlorophyll. Loss of the green color is due to the transformation of pheide *a* to the primary fluorescent chlorophyll catabolite. Aiamla-or et al. [7] reported that chlorophyll-degrading peroxidase 3 was responsible for chlorophyll degradation in postharvest broccoli florets. Although there are data for the spectral absorbance of the enzyme cytochrome *c* oxidase [34,35], we have not found any report on enzymes responsible for the degreening of plants. Although absorbance at 280 nm is used frequently to quantify proteins [36], it is difficult to detect the target enzymes amongst all the proteins. Therefore, spectral reflectance may not directly relate to the velocity of loss of the green color and needs to be tested by statistical methods.

**Figure 5:**
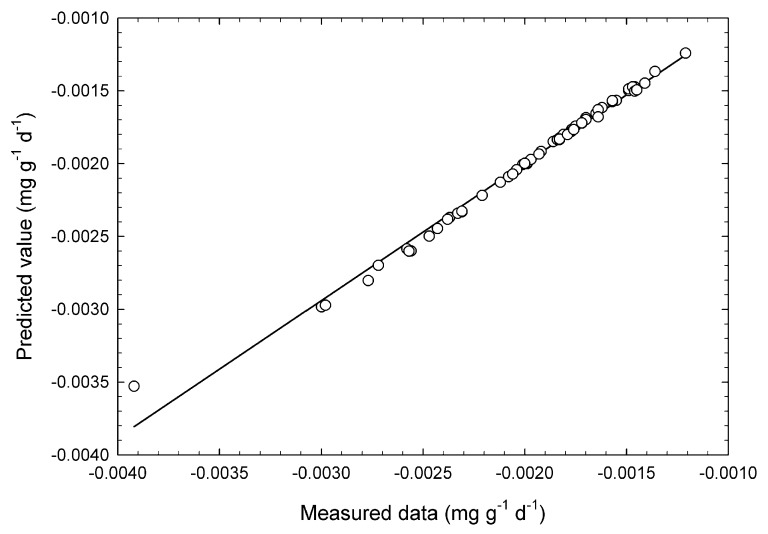
Validation for predicting the chlorophyll degradation velocity of broccoli heads at 60 regions of interest by artificial neural networks. The open circles and full line are the actual values and single regression line. Bias was −4.82 × 10^−7^ mg·g^−1^·d^−1^, standard error of prediction 5.37 × 10^−5^ mg·g^−1^·d^−1^, and correlative coefficient of validation was 0.995. This level of correlation was significant at the 99.9% level calculated using the *F*-test.

The statistics of the calibration and validation data sets are shown in Table 1. The relationship between measured *k* and estimated $\hat{k}$ using ANNs as a measure of validation is shown in Figure 4. The approximation between the values of the proposed equation and the unknown measured values was better with a high correlative coefficient and a low SEP. According to the results from multiple linear regression analysis, bias was 0.377 mg·g^−1^·d^−1^, SEP 6.65 × 10^−3^ mg·g^−1^·d^−1^, and correlative coefficient of validation was 0.247. According to the results from partial least square regression analysis, bias was −1.15 × 10^−4^ mg·g^−1^·d^−1^, SEP 4.48 × 10^−4^ mg·g^−1^·d^−1^, and correlative coefficient of validation was 0.400. Prediction results by these linear regression methods were worse than ANNs as a nonlinear method. Williams & Norris [26] reported that a correlative coefficient over 0.99 could be used in any application. This indicates that ANNs using spectral reflectance from 405 to 960 nm could be utilized for predicting the velocity of chlorophyll reduction. According to the data range in Table 1, ANNs cover the velocity of chlorophyll reduction, which ranged from −3.39 × 10^−3^ to −1.05 × 10^−3^ mg·g^−1^·d^−1^. Therefore, 59 actual data points in Figure 5, which ranged from −3.00 × 10^−3^ to −1.21 × 10^−3^ mg·g^−1^·d^−1^, were supported. Only an actual data as −3.92 × 10^−3^ mg·g^−1^·d^−1^ out of the supported range might be farther from the regression line than the other plots.

**Figure 6:**
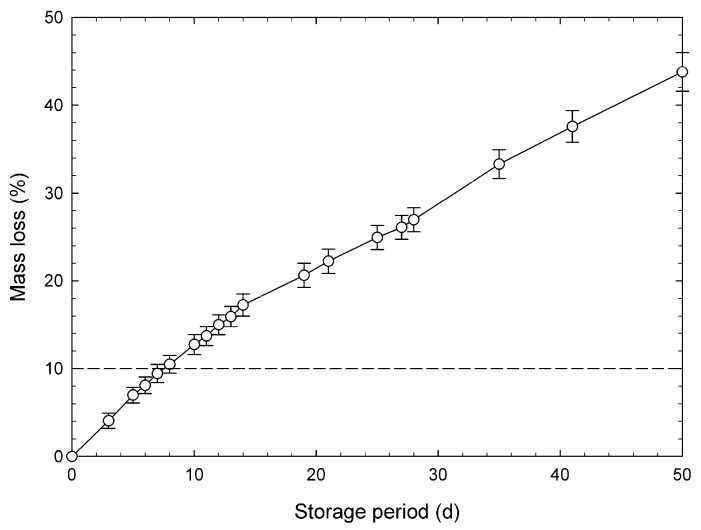
Change in mass loss of broccoli heads. The mean ± SE of six observations has been plotted.

**Figure 7:**
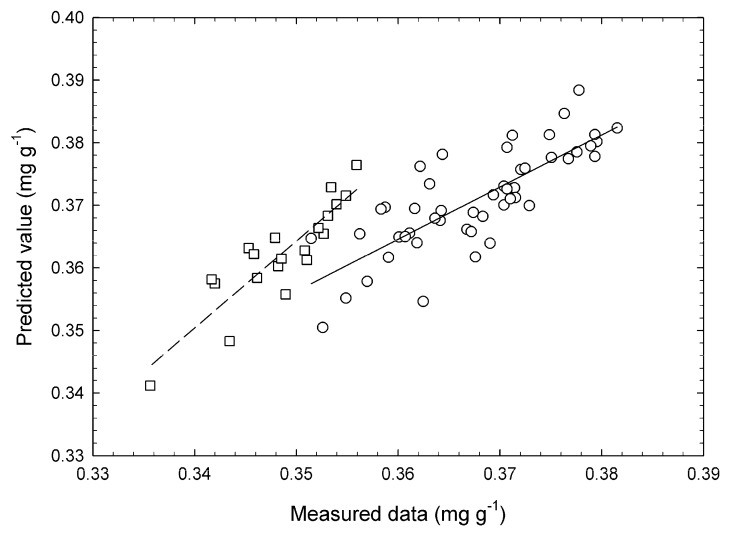
Relationship between measured and predicted chlorophyll concentrations after 3 and 7 d of storage. The circles and squares denote the plots for days 3 and 7, respectively. The solid lines denote the linear regression lines for day 3 (Bias 3.31 × 10^−3^ mg·g^−1^, standard error of prediction 5.03 × 10^−3^ mg·g^−1^·d^−1^, correlative coefficient of validation 0.795; significant at the 99.9% level calculated using the *F*-test). The dashed lines denote the linear regression lines for day 7 (Bias 1.37 × 10^−2^ mg·g^−1^, standard error of prediction 4.33 × 10^−3^ mg·g^−1^·d^−1^, correlative coefficient of validation 0.884; significant at the 99.9% level calculated using the *F*-test).

According to previous reports [37], the *k* values were calculated using a wide range of chlorophyll concentrations, which generated approximated lines or calibration lines using a wide range of data. Although a completely degreened broccoli head is considered to lose its merchantability, there are no reports on the relationship between loss of green color and marketability of the vegetable. Kasim et al. [13] described that a mass loss of 10% makes horticultural products unusable. The changes in mass loss of broccoli heads used for validation are shown in Figure 6. This data shows that mean mass loss exceeded 10% after 7 d of storage and reached 43% at the end of storage. Therefore, the ANN model practically supports the chlorophyll degradation velocity until 7 d. The relationship between measured and predicted chlorophyll concentrations of broccoli buds on day 3 demonstrated that the heads remained fresh and on day 7 had just before lost about 10% of their mass (Figure 7). The predicted values were calculated using $\hat{k}$ values estimated by the constructed ANN model. This may be the reason behind SEP for chlorophyll concentrations being higher than the $\hat{k}$ values. Plots for day 7 tended to shift to the lower left of the figure compared with those for day 3, which is due to the loss of chlorophyll over time. There was a positive correlation between the measured and predicted concentrations. According to the values of the correlative coefficients, this prediction level was effective for rough screening [26]. The proposed ANN model was effective in predicting chlorophyll concentration during storage using the predicted degreening velocity $\hat{k}$.

Reflectance or absorption spectra combined with statistical methods have been used to predict many kinds of characteristics of horticultural products such as TSS, moisture, and hardness [32], and multiple regression analyses or partial least square regression analyses have been adopted as statistical methods [37]. Such linear methods can be adopted to predict contents with a relatively high concentration. However, other methods need to be considered for estimating contents that are difficult to predict using linear methods. ANNs combined with spectral data were evaluated as a potential statistical method [26]. Peiris et al. [32] estimated SSC in apples (*Malus pumila* Mill.), while Makino et al. [38] estimated O_2_ uptake rate of tomatoes (*Solanum lycopersicum* L.). Siripatrawan et al. [39] determined the type and cell number of *Escherichia coli* (Migula) in liquid media and spinach. In the current study, we nondestructively predicted the degreening velocity of broccoli for the first time.

ANNs are useful for fitting to nonlinear relationships [26]. This indicates that there are nonlinear relationships between the velocity of loss of green color and reflectance of visible/near-infrared illumination. Because it is difficult to predict the contents of enzymes, which are directly controlling the chlorophyll degradation, the technique found in this research, a non-linear model combined with the determination of spectral reflectance, may be useful for predicting the velocity of loss of green color, thereby providing a nondestructive prediction of the deterioration velocity of broccoli florets.

## Figures and Tables

**Table 1:** Statistics of exemplar (chlorophyll degradation velocity of broccoli buds) and independent variables (second derivative of absorbance at chosen wavelengths) within calibration and validation sets.

Exemplar or Variables	Calibration Set (*n* = 110)			Validation Set (*n* = 60)		
	Range	Mean	SD ^1^	Range	Mean	SD ^1^
Chlorophyll degradation velocity (mg·g^−1^·d^−1^)	−3.39 × 10^−3^–(−1.05 × 10^−3^)	1.97 × 10^−3^	4.88 × 10^−4^	−3.92 × 10^−3^–(−1.21 × 10^−3^)	1.97 × 10^−3^	4.83 × 10^−4^
405 nm	−2.50 × 10^−5^–1.48 × 10^−5^	−6.60 × 10^−6^	8.38 × 10^−6^	−3.80 × 10^−5^–7.73 × 10^−6^	−1.20 × 10^−5^	8.95 × 10^−6^
420 nm	−1.70 × 10^−5^–5.72 × 10^−6^	−8.40 × 10^−6^	5.09 × 10^−6^	−1.80 × 10^−5^–3.83 × 10^−6^	−7.90 × 10^−6^	5.21 × 10^−6^
440 nm	3.76 × 10^−6^–1.19 × 10^−5^	7.13 × 10^−6^	1.69 × 10^−6^	3.70 × 10^−6^–1.25 × 10^−5^	7.78 × 10^−6^	1.87 × 10^−6^
455 nm	−6.90 × 10^−6^–(−2.90 × 10^−6^)	−4.70 × 10^−6^	8.17 × 10^−7^	−6.30 × 10^−6^–(−3.10 × 10^−6^)	−4.80 × 10^−6^	7.58 × 10^−7^
475 nm	−3.10 × 10^−6^–4.65 × 10^−6^	−5.30 × 10^−7^	1.45 × 10^−6^	−3.20 × 10^−6^–4.71 × 10^−6^	−8.30 × 10^−7^	1.50 × 10^−6^
530 nm	−3.80 × 10^−5^–(−7.40 × 10^−6^)	−1.90 × 10^−5^	5.54 × 10^−6^	−4.10 × 10^−5^–(-8.60 × 10^−6^)	−1.80 × 10^−5^	5.60 × 10^−6^
595 nm	−2.70 × 10^−6^–6.40 × 10^−6^	4.61 × 10^−7^	1.76 × 10^−6^	−2.00 × 10^−6^–5.80 × 10^−6^	8.28 × 10^−7^	1.66 × 10^−6^
605 nm	−2.40 × 10^−5^–(−9.30 × 10^−6^)	−1.6 × 10^−5^	2.64 × 10^−6^	−2.00 × 10^−5^–(-9.50 × 10^−6^)	−1.6 × 10^−5^	2.64 × 10^−6^
615 nm	−3.30 × 10^−7^–2.46 × 10^−6^	1.14 × 10^−6^	6.51 × 10^−7^	−5.70 × 10^−7^–2.76 × 10^−6^	1.19 × 10^−6^	6.71 × 10^−7^
640 nm	2.40 × 10^−6^–9.81 × 10^−6^	6.24 × 10^−6^	1.42 × 10^−6^	2.39 × 10^−6^–9.22 × 10^−6^	6.63 × 10^−6^	1.46 × 10^−6^
670 nm	1.95 × 10^−5^–9.14 × 10^−5^	4.74 × 10^−5^	1.32 × 10^−5^	2.10 × 10^−5^–1.01 × 10^−4^	4.57 × 10^−5^	1.41 × 10^−5^
725 nm	−1.20 × 10^−4^–2.28 × 10^−5^	−4.40 × 10^−5^	2.90 × 10^−5^	−1.40 × 10^−4^–1.66 × 10^−6^	−4.80 × 10^−5^	3.12 × 10^−5^
790 nm	3.68 × 10^−6^–6.55 × 10^−5^	3.67 × 10^−5^	1.55 × 10^−5^	1.14 × 10^−5^–7.99 × 10^−5^	3.91 × 10^−5^	1.54 × 10^−5^
870 nm	−8.90 × 10^−6^–7.72 × 10^−6^	4.00 × 10^−7^	3.53 × 10^−6^	−5.90 × 10^−6^–7.42 × 10^−6^	3.23 × 10^−7^	3.23 × 10^−6^
960 nm	3.61 × 10^−5^–1.07 × 10^−4^	7.14 × 10^−5^	1.51 × 10^−5^	2.79 × 10^−5^–1.02 × 10^−4^	6.97 × 10^−5^	1.85 × 10^−5^

^1^ SD: standard deviation.

## Nomenclature

*a**	color axis of the Commission International de l’Eclairage (CIE)
*A*	constant
*b*	deviation between individual and mean measured degradation velocity of chlorophyll (mg·g^−1^·d^−1^)
*b**	color axis defined by CIE
$\bar{b}$	bias (mean of deviation *b*)
*B*	weighted connection between input and hidden layers in an artificial neural networks
*c*	constant (mg·g^−1^·d^−1^)
*C*	chlorophyll concentration (mg·g^−1^)
*d*	input data: δ^2^ *R* (2nd derivative of spectral reflectance)
*D*	weighted connection between hidden and output layers in an artificial neural networks (mg·g^−1^·d^−1^)
*H*	hidden node in an artificial neural networks
*H°*	hue angle [tan^−1^ (*b**/*a**)]
*k*	measured velocity of chlorophyll degradation (mg·g^−1^·d^−1^)
$\hat{k}$	estimated velocity of chlorophyll degradation (mg·g^−1^·d^−1^)
*m*	total number of independent variables
*n*	total number of samples
*l*	total number of hidden nodes
*R*	spectral reflectance from broccoli buds
*t*	storage time (d)
*X*	sum of weighted input signals into a hidden node
δ	differential operator
*Subscript*	
*i*	a sample
*j*	an independent variable
*h*	a hidden node
*t*	at an arbitrary storage time
0	at the start of storage
